# Everybody's Page

**Published:** 1889-01-19

**Authors:** 


### EVERYBODY'S PAGE.

BRIEF inhalations of carbonic acid gas are said to relieve coughing.

DR. CHARLES W. ALLEN believes that there are at least a hundred and fifty cases of leprosy in the United State, mostly acquired by visiting countries where the disease is prevalent.

OXYPROPRYLENDIISOAMYLAMINE is said to be a heart tonic, but the *New York Medical Record* thinks that it will be most efficacious as a gymnastic exercise in cases of stuttering.

IT has been suggested that croup should be treated with bicarbonate of soda—the "baking soda" of the household—dissolved in water, to which a little milk has been added.

A PHILADELPHIA medical journal says: "The medical legislator is not usually a pleasant figure to contemplate. Is this true? And if true, is it not to be regretted? Most legislatures would be greatly improved by numbering among their members one or two doctors and experts in sanitary science. Unfortunately, very few doctors can afford the time necessary for the performance of legislative duties.

GOOD coffee, by means of its marvellous stimulating influence on the brain, is both the social and the physiological antidote of alcohol. At Rio Janeiro, where the population numbers 500,000, drunkenness is almost unknown, and every-one drinks coffee in large quantities. Even immigrants, who may have brought a love of alcohol with them, end by preferring the delicious coffee which the Brazilians prepare so well.

IF the Morgue at Paris be an institution necessary to the public welfare, as our French neighbours seem to think, it is well that it should not become a danger to public health; and there is therefore matter for some degree of congratulation in the news that the employment of new and improved freezing apparatus has rendered the Morgue, from a sanitary point of view at least, perfectly harmless. The highest temperature in any apartment where corpses are kept is 2 deg. under freezing point, and where the bodies are kept for a longer time, to facilitate legal investigations, an even greater degree of cold is maintained.

HERE is a cheering result of dosimetric treatment in pneumonia. Just eleven medicines were ordered—namely, bromocamphor, dose of 8 granules; genuine hydrobromate, 4; arbutine, 1; strychnine hypophosphate, 1; aconitine, 1; veratrine, 1; digitaline, 1; sodium benzoate, 1; iodoform, 1; hellenine, 1; musk, 1. Each of these was to be taken once in two hours; that is, a new medicine every ten minutes. To make up the dozen two granules of quassine were to be administered every time the patient took any food. The treatment went on for ten days. Then the patient died, possibly from the difficulty, not to say impossibility, of remembering which medicine came next.

IN the summer of 1884 an epidemic of diphtheria broke out in Skiatos, a small island in the Grecian archipelago. No case of that disease having occurred on the island for more than thirty years, some curiosity was felt as to how the epidemic originated, and various inquiries and investigations pointed to the conclusion that it was caused by a flock of turkeys recently imported from Salonica, some of which were ill when they arrived, while the others sickened afterwards from a disease of a diphtheritic nature. There had been no direct contact between the fowls and the children first attacked, but both were in the same quarter of the town, and the infection seemed to have been carried by the wind.

MANY districts of Algeria, once deadly, have been rendered healthy by the planting of Eucalyptus trees, and it is probable that by this means the Roman Campagna, now desolate and malarious, could be reclaimed and made habitable.

The remedial action of drugs at a distance, one of the latest varieties of the hypnotic craze, reminds one of the "sympathetic ointment" of old, in which Sir Kenelin Digby believed so firmly. If you were wounded by sword or bullet, a linen rag soaked in cold water was put on the wound; but nobody expected that to do any good. Then the weapon that did the wrong, if attainable, was dressed with the ointment and carefully bandaged. It was then left for ten days, and if at the end of that time your wound was cured or much improved, everybody exclaimed at the wonderful power of the ointment, and nobody thought the least credit was due to the linen rag and the cold water that soothed the injury itself.

WHAT is the cause of consumption? This is a question difficult to answer, but an American lady who goes in for "mind-curing" has a theory of her own. "Consumption," she says, "has its basis in home sickness—longing for home-ties. It repeats itself through fear and a belief in it. Fear always indicates a vacuum." She also has original views on strychnia, and thus explains why the mind-curers cannot prevent poisoning by that drug: "No man stands alone. You are a part of me. Until your thought reaches a point at one with my thought I am found by you. As long as you hold to the belief that strychnia is poison, how am I going to be strong enough to overcome it?" This is ingenious, but not wholly satisfactory.

A UNIQUE remedy for eczema and other cutaneous diseases is Hebra's water-bed; or rather, to define it accurately, continual bath. The patient lives entirely in his bath, eats, drinks, and sleeps there till he is well. One patient was kept in such a bath for 385 days. Think of this! more than a year of washing-day! It is enough to make anyone hate the sight of water for ever. There is a slight danger of the patient slipping entirely under water in his sleep, and drowning; but it is usual to pass a bandage under his arms and fasten it, so as to support the upper part of the body on the inclined part of the bath on which the head rests. The palms and soles become white and shrivelled, like a washerwoman's hands, but the skin of other parts of the body does not suffer, and the treatment is said to be very efficacious in severe burns. When the patient first gets into the bed the temperature is 86 deg., but as he gets used to it it is raised by degrees to 98 deg.

MR. L. WEBSTER has suggested the purification of sewage by electricity. His experiments prove that by passing an electric current through a thick, heavy, and offensive liquid taken from a London drain, a movement is brought about which causes the solid particles to separate from the liquid that has hitherto carried them. In fifteen minutes or so these solid particles accumulate in a scum at the top, which can be easily taken off, leaving the water clear and free from odour. The reason that the solids float on the surface of the water, in apparent opposition to the laws of gravity, is that the gases which are set free by the reaction caused by the electric current, mingle with them and make the total mass lighter than the liquid in which they formerly floated. Mr. Webster has calculated that this treatment is less costly than the suggested purification of sewage by salts of iron, and that the residue would be more suitable for use in agriculture.

